# Limited Variation in Bacterial Communities of *Scaphoideus titanus* (Hemiptera: Cicadellidae) Across European Populations and Different Life Stages

**DOI:** 10.3390/insects15110830

**Published:** 2024-10-23

**Authors:** Juan Sebastian Enciso, Erika Corretto, Luigimaria Borruso, Hannes Schuler

**Affiliations:** 1 Faculty of Agricultural, Environmental and Food Sciences, Free University of Bozen-Bolzano, 39100 Bolzano, Italy; jencisogarcia@unibz.it (J.S.E.); luigimaria.borruso@unibz.it (L.B.); 2 Competence Center for Plant Health, Free University of Bozen-Bolzano, 39100 Bolzano, Italy; erika.corretto@unibz.it

**Keywords:** *Scaphoideus titanus*, bacterial communities, 16S rRNA gene metabarcoding, endosymbionts, *Karelsulcia*, *Cardinium*

## Abstract

The Nearctic leafhopper *Scaphoideus titanus* (Hemiptera: Cicadellidae) is the primary vector of ‘*Candidatus* Phytoplasma vitis’, the causative agent of Flavescence doreé in Europe. Although microorganisms play an important role in the ecology and behavior of insects, knowledge about the interaction between *S. titanus* and microbes is limited. In this study, we employed an amplicon metabarcoding approach for profiling the V4 region of the 16S rRNA gene to characterize the bacterial communities of *S. titanus* across several populations from four European localities. Additionally, we investigated changes in bacterial communities between nymphal and adult stages. In total, we identified 7,472 amplicon sequence variants (ASVs) in adults from the European populations. At the genus level, ‘*Candidatus* Karelsulcia’ and ‘*Candidatus* Cardinium’ were the most abundant genera, with both being present in every individual. While we found significant changes in the microbial composition of *S. titanus* across different European populations, no significant differences were observed between nymphal and adult stages. Our study reveals new insights into the microbial composition of *S. titanus* and highlights the role of geography in influencing its bacterial community.

## Simple Summary

*Scaphoideus titanus* is the main transmission vector Flavescence doreé (FD) in Europe, causing significant damage to grapevine production. The causative agent of FD is the bacterium ‘*Candidatus* Phytoplasma vitis’. However, little is known about the interaction of this plant pathogen with the bacterial communities within the insect host. In this study, we characterize the bacterial communities in *S. titanus* across different European populations and different life stages. We found significant differences in the microbial composition across different populations but did not observe any differences between nymphs and adults of the same population.

## 1. Introduction

Phytoplasmas are important plant pathogens which are mainly transmitted by sap-feeding hemipterans [1]. In Europe, the Nearctic leafhopper *Scaphoideus titanus* Ball (Hemiptera: Auchenorrhyncha: Cicadellidae) is the most important vector of ‘*Candidatus* Phytoplasma vitis’ in grapevines, which is associated with the grapevine yellow Flavescence doreé [2]. The native distribution range of *S. titanus* in North America covers Midwestern and Eastern United States and Canada [3]. In the 1960s, *S. titanus* was introduced in Europe, in France [4], and subsequently invaded 18 European countries, from the Russian Caucasus to Portugal [3], including European territories overseas like Madeira [5].

In the order Hemiptera, plant sap feeders are inherently dependent on symbiotic microorganisms, which offset the lack of amino acids in their diet [6]. These microorganisms are known as primary endosymbionts and are widespread in herbivorous hemipteran species [7]. Hemipterans often maintain one or more essential symbiotic bacteria, such as ‘*Candidatus* Buchnera aphidicola’ in aphids [8], ‘*Candidatus* Carsonella ruddii’ in psyllids [9], and ‘*Candidatus* Portiera aleyrodidarum’ in whiteflies [10]. These symbionts are housed in specialized organs of the insect host called bacteriomes [11]. In most Auchenorrhyncha, the suborder of Hemiptera that includes cicadas, leafhoppers, planthoppers, and treehoppers, at least two symbionts co-occur within one host species. The Bacteroidota ’*Candidatus* Karelsulcia muelleri‘ (hereafter *Karelsulcia*) is present in most Auchenorrhyncha and has been associated with its host for about 270 million years [12]. However, symbiotic associations are not always stable, and occasional losses and replacements can occur, as shown in Philaenini spittlebugs [13] and various lineages of cicadas [14]. While *Karelsulcia* provides most of the 10 essential amino acids (EAAs), a co-occurring Betaproteobacteria or Gammaproteobacteria symbiont synthesizes the remaining ones [15,16]. In Deltocephalinae leafhoppers, *Karelsulcia* co-occurs with ‘*Candidatus* Nasuia deltocephalinicola’ [17] and occasionally with *Sodalis*, where triple/quadruple associations, e.g., *Karelsulcia–Nasuia–Arsenophonus–Sodalis* have been observed [18]. Additionally, some lineages have lost the Betaproteobacteria symbiont and acquired a yeast-like symbiont [19].

Apart from the primary symbionts, hemipterans harbor a variety of other secondary symbionts that are not essential for host survival [20]. In leafhoppers, common facultative symbionts are the Alphaproteobacteria *Wolbachia* [21] and *Rickettsia* [22], as well as the Bacteroidota *Cardinium* [23]. The effect of these symbionts on the insect host are broad and can range from negative effects such as feminization of genetic males [24] and cytoplasmatic incompatibility between female and males [25] to positive effects such as increasing the lifespan of the host [26] and the defense from natural enemies [27]. The associations of insects with secondary symbionts are much more dynamic than the intimate relationship with primary symbionts [28]. While facultative symbionts have evolved ways to propagate within a host population, affecting the reproduction of its host [29], they can also be transmitted horizontally among species when occupying the same ecological niche [30], or they can be acquired from the diet, via plant sap feeding [31]. Moreover, the composition of the microbiome can change during the host different developmental stages [32].

In *S. titanus, Cardinium* was reported in female reproductive tissues and embryos [33]. Although it is generally assumed that *Cardinium* is not a nutritional symbiont, a biotin pathway was described in the genome of *Cardinium* in *Encarsia pergandiella* (Hymenoptera: Aphelinidae) [34], suggesting a possible contribution to the nutrition of the host. The distribution pattern of *Cardinium* reveals a wide range of invertebrate hosts, including arthropods [35,36] and nematodes [37]. *Cardinium* can be transmitted vertically and induce cytoplasmatic incompatibility in crosses where males are infected with the symbiont and females are not, thus promoting its own spread [38]. Molecular studies have reported that *S. titanus* harbors other symbionts, like *Asaia* [39] and various other bacteria that are considered grapevine endophytes, such as *Agrobacterium*, *Pseudomonas*, *Erwinia*, *Paracoccus*, and *Lysobacter* [40].

Despite the understanding of the microorganisms associated with *S. titanus* has increased in recent years [40,41], comprehensive knowledge of the taxonomic composition across populations is limited. Moreover, knowledge of the diversity of the bacterial communities associated with *S. titanus* across different life stages is lacking. Therefore, we characterize the bacterial community composition of *S. titanus* across four European countries and compare the bacterial composition and diversity among different life stages.

## 2. Materials and Methods

### 2.1. Sample Collection and Identification

In total, we analyzed 45 individuals of *Scaphoideus titanus*: 40 adults and five nymphs (Table 1). To investigate the bacterial community across different geographic regions, we analyzed eight adults from Bordeaux, France (Fr); ten from Breganze, Italy (It); eight from Brno, Czech Republic (Cz); seven from Malca, Serbia (Sr1) and seven from Belgrade, Serbia (Sr2). To compare the microbial composition among different life stages, we characterized the microbial community of five nymphs (fifth stage) and compared them with adults from the same locality (Fr1). All samples were collected between August 2020 and May 2023. Individuals of *S. titanus* from Italy, Czech Republic, and Serbia were collected by using sticky traps and sweeping nets in vineyards, placed immediately in absolute ethanol and stored at −20 °C. Samples from France were obtained by collecting logs containing *S. titanus* eggs during winter, which were stored at 4–8 °C in a cold chamber. Egg hatchings were obtained by placing the logs in a climate chamber at 22 °C, 16:8 (L:D) photoperiod, and 65–70% relative humidity, as described in [42]. Nymphs were reared on broad beans, and after completing development, young adults were collected, placed in absolute ethanol, and stored at −20 °C.

### 2.2. DNA Extraction and Sequencing

DNA was extracted by using DNeasy Blood & Tissue Extraction Kit (Qiagen, Hilden, Germany) according to the manufacturer’s protocol without surface sterilization of the insect. DNA quality and quantity were measured with the fluorometer DS-11 FX+ (Denovix Inc., Wilmington, DE, USA) and the Qubit 1X dsDNA High Sensitivity (HS) kit (Invitrogen, Waltham, WI, USA).

Taxonomic identification was verified by performing molecular barcoding targeting the *cytochrome oxidase* II (COXII) gene. PCR was performed by using the primers mtd13 and mtd18 [43]. PCR reactions were performed in a total volume of 25 µL containing 2 µL of DNA, 0.5 µL of each primer (10 µM), 12.5 µL of 2X DreamTaq Master Mix (ThermoFisher, Waltham, USA), and 9.5 µL of sterile water under the following thermal conditions: 3 min at 95 °C for initial denaturation; 35 cycles of 30 s at 95 °C; 40 s at 50 °C, 1 min at 72 °C, and 10 min at 72 °C. PCR products were Sanger-sequenced at Eurofins Genomics (Ebersberg, Germany) and then analyzed by BLAST search [44] by using the NCBI database.

Bacterial communities were characterized by performing the amplicon sequencing of the V4 hypervariable region of the 16S rRNA gene by using the barcoded primers 515f and 806r [45]. Sequencing was carried out on an Illumina MiSeq with 250 bp paired-end reads at StarSEQ GmbH (Mainz, Germany). Additionally, a no-template control was included as the negative control.

### 2.3. Data Analysis

Raw reads were processed by using the QIIME2 pipeline (version 2023.9); [46]. Due to the poor quality of the reverse reads, only the forward reads were used in this study. However, because of the short size of the V4 region of the 16S rRNA gene, the forward reads covered almost the complete gene. The trimming of the forward reads was performed at 230 bp by using the DADA2 plugin in q2-dada2. DADA2 was also used for the clustering and denoising of the raw reads into amplicon sequence variants (ASVs) [47]. Taxonomic classification was performed by using the SILVA database, version 138 [48]. Subsequently, ASVs belonging to archaea, mitochondria, eukaryotes, and chloroplast, as well as rare ASVs (i.e., singletons), were removed. Additionally, ASVs that were present in the no-template control were excluded from the analyses. Unclassified ASVs at the genus level were further investigated by using BLAST search [44] and the NCBI database. The two datasets were analyzed independently; therefore, two ASV tables were generated: one for the microbiome characterization of adults from different European populations (Table S1) and a second ASV table for the comparison of two life stages of one locality in France (Table S2). Raw reads were submitted to NCBI under BioProject PRJNA1155196.

### 2.4. Statistical Analyses

Statistical analyses were performed in R (Version 4.3.2). The packages ‘phyloseq’ [49] and ‘vegan’ [50] were used for assessing bacterial diversity. For alpha diversity, the number of observed ASVs and the Chao1, Evenness, and Shannon indices were calculated. To test the effect of the sampling site on the Chao1, Evenness, and Shannon indices, we used the one-factor analysis of variance (ANOVA) test. Tukey’s honestly significant difference test was implemented as a post hoc test to show the differences among the populations of different localities. In addition, rarefaction was performed to standardize the sequencing depth across samples, ensuring comparability by subsampling each sample to the same number of reads. To visualize the relative abundance of the observed taxa, we used the ‘ggplot’ package in R [51]. A heatmap was generated by using the ‘pheatmap’ package in R [52] to visualize the relative abundance across the 30 most abundant taxa by using log 2 transformation.

To calculate beta diversity and relative abundance of the data, reads were normalized by dividing the reads of each ASV by the total count within each sample. Beta diversity was assessed by using Bray–Curtis distances matrices. The ordination of the Bray–Curtis matrices was visualized by using principal coordinates analysis (PCoA) performed with the ‘ordinate’ function from ‘phyloseq’ [49]. PCoA plots were generated to visualize the clustering of individuals based on locality and life stage. To statistically test for differences in bacterial composition among groups, a PERMANOVA (Permutational Multivariate Analysis of Variance) was conducted by using the ‘adonis2’ function from the ‘vegan’ package. The analysis was based on the Bray–Curtis distance matrix, with 9999 permutations testing for significant composition across different life stages and populations.

## 3. Results

### 3.1. Bacterial Communities of S. titanus from Different European Populations

The molecular barcoding of all individuals confirmed that all the specimens used in this study were correctly identified as *S. titanus*. A total of 40 adults of *S. titanus* from different populations (Fr, It, Cz, Sr1, and Sr2) produced a total of 2,880,698 reads with an average of 72,017 ± 16,416 reads per individual, ranging from 30,279 to 102,053 reads per individual. After filtering and trimming, the remaining reads were assigned to 52 bacterial phyla (Table S1).

**Figure 1:**
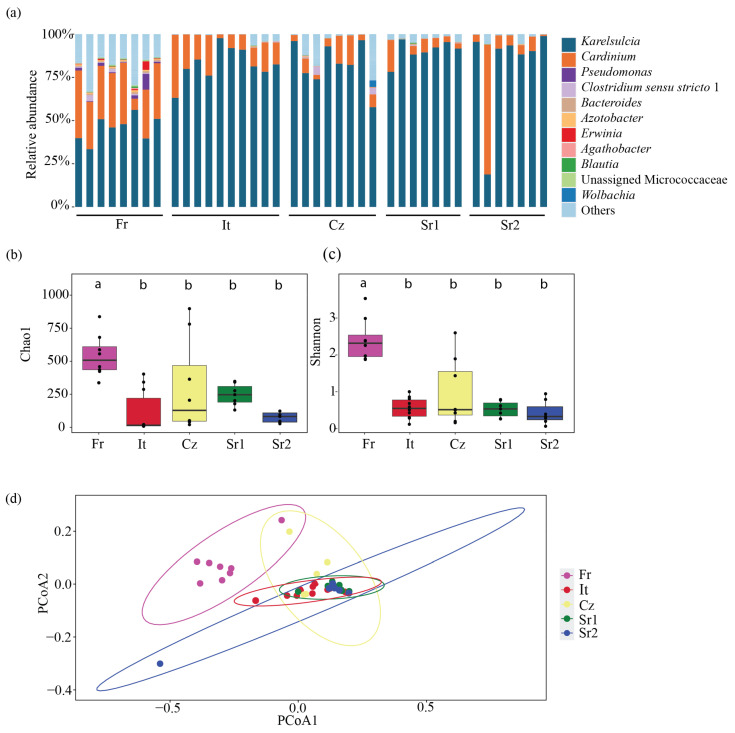
(Bacterial community composition and diversity in adults of *Scaphoideus titanus*. (**a**) Relative abundance at genus level. (**b**) Comparison of bacterial species richness based on Chao1 index of adults in *S. titanus*. (**c**) Comparison of bacterial diversity based on Shannon index of adults in *S. titanus*. (**d**) Principal coordinates analysis based on Bray–Curtis dissimilarity matrix showing differences among individuals across Europe. Different letters represent statistically significant differences.

At the genus level, 11 bacterial taxa were predominantly found in adults from different European populations: *Karelsulcia*, *Cardinium*, *Pseudomonas*, *Clostridium sensu stricto 1*, *Bacteroides*, *Azotobacter*, *Erwinia*, *Agathobacter*, *Blautia*, Unassigned Microccocaceae, and *Wolbachia* (Figure 1a). *Karelsulcia* was detected in all samples, representing 78.3% of all reads. The second most abundant ASV was assigned to *Cardinium*, representing 13.5% of all reads. The highest relative abundance of *Cardinium* was detected in one individual from Serbia (Sr2) with a relative abundance of 75.21%, while individuals from Czech Republic (Cz) and Serbia (Sr1 and Sr2) and one individual from Italy (It) had low relative abundance, ranging from 0.07 to 2.43%.

**Figure 2:**
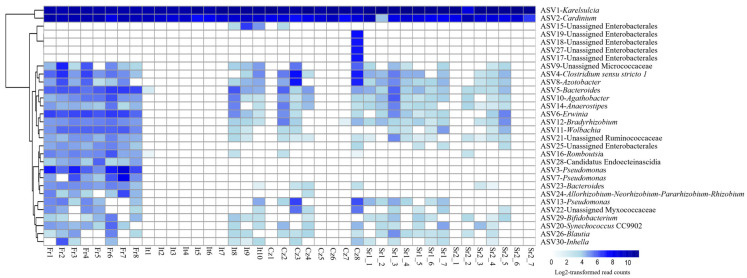
Heatmap showing the distribution of the 30 most abundant amplicon sequence variants (ASVs) in adult individuals of *Scaphoideus titanus* across Europe.

While *Karelsulcia* and *Cardinium* represented together 91% of all reads, all other ASVs were present with only low abundance rates, less than 1% of all reads each. Interestingly, these low-abundance ASVs were not equally distributed among the different individuals and populations (Figure 2). The individuals from the Fr group harbored several other taxa with higher relative abundance compared with the other populations. In particular, seven individuals from Italy (It), three from Czech Republic (Cz), and three individuals from Serbia (Sr2) had only reads belonging to *Karelsulcia* and *Cardinium*. Among the low-abundance taxa, *Clostridium sensu stricto 1* was present in 65% of the samples. Furthermore, a BLAST search on four unidentified ASVs at the genus level showed that Unassigned Enterobacterales (ASV6) was identical to *Erwinia* sp. (KY856906) and Unassigned Pseudomonadaceae (ASV8) was identical to *Azotobacter* sp. (MN853548). ASV24, which was classified as Unidentified Myxococcaceae, showed 95% similarity with different genera, such as *Corallococcus*, *Archangium*, and *Cytobacter*. Two ASVs were classified as *Pseudomonas* (ASV3 and ASV7) and were exclusively found in adults from the Fr population. A third ASV13-*Pseudomonas* was present in individuals from the It and Sr1 groups (Figure 2).

Bacterial species richness across the different populations was relatively low, ranging between 10 and 975 ASVs (Table S2). At the population level, the bacterial richness was higher among the Fr population, ranging between 423 and 975 ASVs, followed by the Cz population, with 20 to 898 ASVs, and the Sr1 population, with 131 to 348 ASVs. The It population had the lowest species richness, ranging from 8 to 403 ASVs. Moreover, bacterial species richness and diversity based on the Chao1 and Shannon indices showed significant differences among sampling sites, especially between the Fr population and the other localities (Chao1: ANOVA, F = 6.92, df = 4, *p* < 0.001; Shannon: ANOVA, F = 19.68, df = 4, *p* < 0.001) (Figure 1bc, Table S3). Additionally, we performed a principal coordinates analysis (PCoA) using Bray–Curtis dissimilarities to compare beta diversity in the different populations. This analysis revealed two major clusters: one comprising individuals from the Fr population and the other containing the remaining European populations (PERMANOVA, F = 8.39, df = 4, R^2^ = 0.48972, *p* < 0.001) (Figure 1d).

### 3.2. Bacterial Community of S. titanus Across Different Life Stages

**Figure 3:**
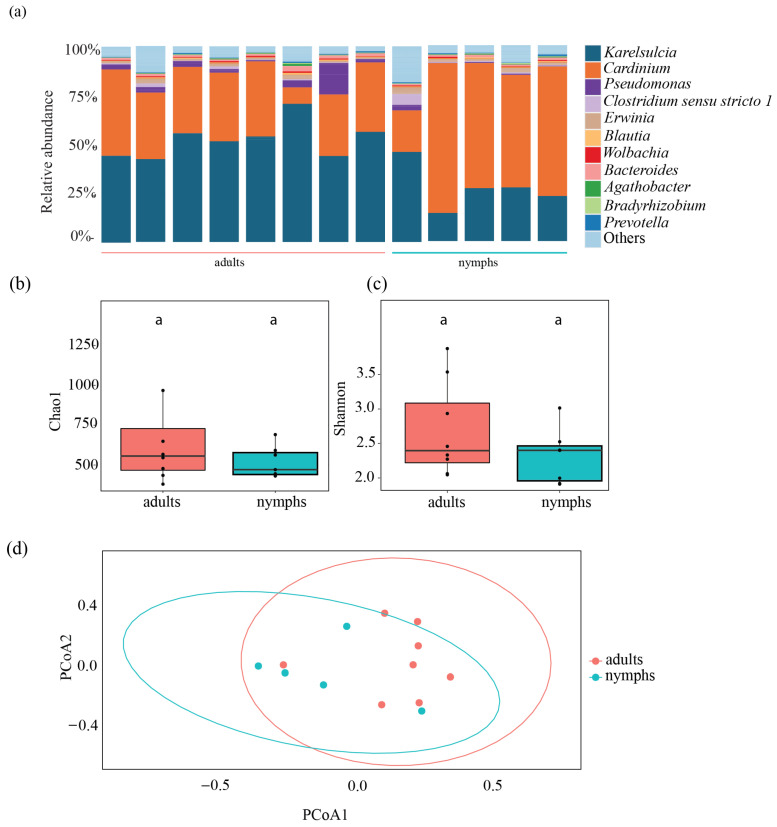
Bacterial composition of the most common taxa detected in nymphs and adults of *Scaphoideus titanus* from France. (**a**) Relative abundance at genus level. Comparison of bacterial species richness based on Chao1 (**b**) and Shannon indices (**c**) of adults and nymphs in *S. titanus*. (**d**) Principal coordinates analysis based on Bray–Curtis dissimilarity matrix showing Shannon diversity index of nymphs and adults from Bordeaux. Different letters represent statistically significant differences.

To assess differences between the different life stages, we analyzed five nymphs and compared them to eight adults from the Fr population. We obtained a total of 496,972 reads, which ranged from 61,836 to 105,982 reads per individual. *Karelsulcia* was found across both life stages, in nymphs (14,173 ± 4666 reads) and in adults (28,440 ± 4326 reads) (*t*-test: *p* < 0.001), with lower relative abundance in nymphs (12.63–35.64%) compared with adults (34.01–56.68%) (Figure 3a). In contrast, *Cardinium* showed a higher relative abundance in nymphs (30,307 ± 10,804 reads) compared with adults (18,053 ± 5710 reads) (*t*-test: *p* = 0.08). The third most abundant taxon was *Pseudomonas*, which was present in low abundance in every adult but only in some nymphs. Additionally, *Bacteroides* were detected in adults and nymphs (Figure 3a).

Overall, there were no significant differences between nymphs and adults in terms of species richness, diversity, and evenness (Chao1: ANOVA, F = 1.604; df = 1, *p* = 0.227; Shannon: ANOVA, F = 0.785; df = 1, *p* = 0.395; Pioulu’s: ANOVA, F = 0.482; df = 1, *p* = 0.502). The Chao1 index in adults had a median value of 606.12 compared with 576.28 in nymphs (Figure 3b, Table S3), while Shannon diversity values for adults and nymphs were 2.56 and 2.45, respectively (Figure 3c, Table S3). The PCoA showed no clear segregation between nymphs and adults (PERMANOVA, df = 1, F = 2.2865, R^2^ = 0.172, *p* = 0.7; Figure 3d).

## 4. Discussion

The leafhopper *Scaphoideus titanus* is the most important vector of Flavescence dorée phytoplasma and represents one of the biggest threats to grapevine production in Europe [3,53]. Therefore, advances in understanding the relationship between this pest species and its associated microorganisms are crucial to getting a clear picture of its biology. Since symbionts have been proposed as ecological alternatives to control insect pests and their associated pathogens, knowledge about these organisms is key to developing effective symbiont-based application strategies [54,55].

Here, we characterized the bacterial community composition of *S. titanus* across Europe. While previous studies used pools of multiple individuals [40,41], our single-individual-based approach showed a predominance of two bacterial symbionts, *Karelsulcia* and *Cardinium*, in every specimen of *S. titanus*. *Karelsulcia* has been already described in *S. titanus* in the United States and in the invasive range in France and Italy [41]. This confirms the importance of this symbiont in this species due to its role in the supplementation of essential amino acids [12]. As shown in previous studies in *S. titanus* [33,41,56], *Cardinium* is commonly found in all populations across Europe. Every single individual analyzed harbored this symbiont, thus suggesting that *Cardinium* might be considered part of the core microbiome of *S. titanus* in Europe. While this bacterium is generally considered a facultative symbiont and interferes with the host’s reproduction, its function in *S. titanus* needs to be further investigated. Interestingly, the only study that analyzed a few individuals from a native population from the United States did not find *Cardinium* [41]. It is, therefore, possible that *S. titanus* acquired *Cardinium* after its introduction, as it was shown for *Rickettsia* in white flies [57] and *Wolbachia* in tephritid fruit flies [30].

Apart from these two most common bacterial taxa, various additional bacteria were found in different localities across Europe with different frequencies. For instance, *Pseudomonas* and *Clostridium sensu stricto 1* were the most abundant taxa after *Karelsulcia* and *Cardinium. Pseudomonas* was specifically found in adults from Bordeaux, France. This genus was also detected previously in nymphs of *S. titanus* occurring in the Trentino region (Italy) that fed on grapevine stems and roots [40]. *Pseudomonas* is a common bacterium found in the grapevine xylem sap and in sharpshooters that feed on these plants [58]. Members of this genus were shown to promote the fitness of their host in plants [59] and insects [60]. In the brown planthopper *Nilaparvata lugens*, this bacterium contributes to better tolerance of adverse climate conditions such as temperature and draught [61]. *Clostridium sensu stricto 1* is a spore-forming bacterium commonly found in soil, water, and plants [62]. In grapevines, this genus occurred in the rhizosphere and in the flowering and early fruiting stages [63], suggesting that it might be acquired by *S. titanus* by feeding on the plant. Other taxa that were common in *S. titanus* were *Azotobacter*, Unassigned Enterobacterales, *Agathobacter*, and *Bacteroides,* which are part of the soil microbiome [64]. Although the Alphaproteobacteria *Asaia* was described to be associated to *S. titanus* from the Italian regions of Trentino and Piedmont [40,65], this genus was not detected in our dataset. Interestingly, it was shown that *Asaia* can interfere with *Phytoplasma* transmission in *S. titanus* individuals and thus might be a candidate for a future biological control agent [39].

Overall, the bacterial communities across the different populations in Europe were very homogenous. However, specimens collected from France showed a significantly different bacterial composition. While all the other insect samples were collected in summer in the field, the samples from France derived from grapevine logs harboring *S. titanus* eggs which had been kept overwintering under laboratory conditions and emerged in the laboratory in the following year. However, it is not clear why the samples collected as adults in nature show lower bacterial diversity than individuals collected as eggs and treated in generally more sterile conditions in the lab. Since the feeding habits can influence or change the microbial composition of the host [40,66] the use of broad bean plants as feeding plants for rearing might have influenced the bacterial community of *S. titanus*.

The ontogenetic development of insects alters the structure of bacterial communities [67]. For holometabolous insects such as fruit flies [68] and bark beetles [69], changes in the bacterial communities are very common between the different developmental stages. Changes in bacterial communities were also observed in hemimetabolous insects. In the brown planthopper *N. lugens*, significant changes in bacterial communities were described across different life stages. Especially, the relative abundance of the phyla Actinomycetota and Bacteroidota decreased through the life stages, showing lower abundance in the adult stages [70]. This contrasts with our study, where we did not find any significant differences in the microbial community of *S. titanus* across two life stages.

## 5. Conclusions

In this study, we characterized the bacterial community of *S. titanus* in four European countries and across different life stages. We found that *Karelsulcia* and *Cardinium* were the predominant taxa of the microbial communities in *S. titanus* present in all individuals and populations across the four European countries. While the omnipresence of *Karelsulcia* is not surprising, being a primary symbiont of almost every Deltocephalinae leafhopper, the presence of *Cardinium* in every single individual is remarkable. Especially the function and role of *Cardinium,* which is generally considered a facultative endosymbiont in other insects, needs to be investigated in future studies.

## Figures and Tables

**Table 1:** List of all sampling locations of *Scaphoideus titanus*, including population code, coordinates, number of analyzed individuals, life stage, and date of collection. * Individuals from France were obtained from a rearing laboratory as described in [42].

Country	Locality	Population Code	Coordinates	Date of Collection	Life Stage	Samples	Number of Individuals
France	Burgundy *	Fr	-	May 2023	Adult	Fr (1–8)	8
					Nymph	Fr (9–13)	5
Italy	Breganze, Veneto	It	45°42′37.44″ N 11°32′50.57″ E	August 2021	Adult	It (1–10)	10
Czech Republic	Brno, Moravia	Cz	48°44′24″ N 16°44′28″ E	August 2020	Adult	Cz (1–8)	8
Serbia	Nis, Malca	Sr1	43°20′46.31″ N 22°2′19.35″ E	August 2020	Adult	Sr1 (1–7)	7
	Belgrade	Sr2	44°51′19.3″ N 20°22′39.1″ E	August 2020	Adult	Sr2 (1–7)	7

## Data Availability

The data used in this study are available at NCBI under BioProject PRJNA1155196.

## Supplementary Materials

The following supporting information can be downloaded at: https://www.mdpi.com/article/10.3390/insects15110830/s1, Table S1: ASV table of adults from different populations of *S. titanus* in Europe, Table S2: ASV table of adults and nymphs from France (Fr), Table S3: Alpha diversity analysis of *S. titanus* from adults across Europe and nymphs in France (Fr).
